# Recommendations for reporting histopathology studies: a proposal

**DOI:** 10.1007/s00428-015-1762-3

**Published:** 2015-04-07

**Authors:** N. Knijn, F. Simmer, I. D. Nagtegaal

**Affiliations:** Department of Pathology, Radboud University Medical Center, PO Box 9101, 6500 HB Nijmegen, The Netherlands

## Background

Most published histopathology studies (describing histological characteristics of existing or new entities, existing or new markers detected by immunohistochemistry, in situ hybridization or molecular methods in tumor material often in relation to patient outcome) are retrospective and use tissue samples from a single center only. This limits the quality of the evidence provided in such a paper. A higher level of evidence, such as would be required to justify implementation in daily clinical practice, can be reached for tissue-based biomarkers by systematic review of published studies and meta-analysis of the provided data.

In such meta-analyses, only research data of sufficient quality should be used. Universally accepted criteria for the assessment of data quality do not exist. However, an essential element would be reporting at a sufficient level of detail of the key components that make up the body of evidence presented in any particular paper. This would also facilitate repetition of the experiments performed and of the relevant observations, an essential step as reproducibility is an absolute prerequisite for validation of tissue biomarkers prior to their implementation in clinical practice.

For in situ hybridization and immunohistochemistry biomarkers, the minimum information specification for in situ hybridization and immunohistochemistry experiments (MISFISHIE) guidelines have been developed to ensure that a report contains sufficient detail of the assay used [1]. MISFISHIE guidelines identify six types of information to be provided for each experiment: experimental design, biomaterials (biospecimens used) and treatments (preanalytical conditions such as fixation and embedding), reporters (antibodies and probes), staining (fluorescence or chromogenic), imaging data (how images were obtained), and image characterization (how information was extracted from the images, including quantification of relevant image elements). However, they do not focus on statistics (correlation of image-derived information with clinical data) or interpretation of the results, which are essential elements of a scientific paper.

To improve possibilities to compare results across studies involving molecular prognostic biomarkers, the Reporting Recommendations for Tumor Marker Prognostic Studies (REMARK) guidelines have been developed. These are intended to facilitate evaluation of the appropriateness and quality of study design, used methods, approaches applied to data analysis, and presentation of the results [2]. The REMARK guidelines can also be used for the reporting of biomarker studies that are not strictly molecular, such as those reporting retrospective histopathological observations, although some items on the checklist will then be less applicable. Notably, the building of prognostic models, checking model assumptions, model validation, and internal validation might not be feasible.

In view of a perceived need for better standardization of retrospective histopathology studies, we have used the REMARK guidelines as a blueprint for the development of basic rules for their reporting [3]. In analogy with the REMARK guidelines, we propose a checklist of 20 items, grouped according to the generally used headings in a research paper: Introduction, Material and methods, Results, and Discussion. We have put these together in a table and will discuss each of them briefly. The intention of our commentary is to increase awareness of the need for more standardization and to stimulate discussion, in order to get to a generally accepted approach to standardized reporting of histopathological studies (Table 1).

**Table 1:** Proposed items for reporting histopathology studies

Introduction
1. State the marker of interest, the study objectives, and hypotheses
Material and methods
2. Describe patient characteristics, and inclusion and exclusion criteria
3. Describe treatment details
4. Describe the type of material used
5. Specify how expression of the biomarker was assessed
6. Describe the number of independent (blinded) scorers and how they scored
7. State the method of case selection, study design, origin of the cases, and time frame
8. Describe the end of the follow-up period and median follow-up time
9. Define all clinical endpoints examined
10. Specify all applied statistical methods
11. Describe how interactions with other clinical/pathological factors were analyzed
Results
12. Describe the number of patients included in the analysis and reason for dropout
13. Report patient/disease characteristics (including the biomarker of interest) with the number of missing values
14. Describe the interaction of the biomarker of interest with established prognostic variables
15. Include at least 90 % of initial cases included in univariate and multivariate analyses
16. Report the estimated effect (relative risk/odds ratio, confidence interval, and *p* value) in univariate analysis
17. Report the estimated effect (hazard rate/odds ratio, confidence interval, and *p* value) in multivariate analysis
18. Report the estimated effects (hazard ratio/odds ratio, confidence interval, and *p* value) of other prognostic factors included in multivariate analysis
Discussion
19. Interpret the results in context of the working hypothesis elaborated in the introduction and other relevant studies; include a discussion of limitations of the study.
20. Discuss potential clinical applications and implications for future research

*FOI* factor of interest, *RR* relative risk, *OR* odds ratio, *CI* confidence interval, *HR* hazard ratio, *UV* univariate, *MV* multivariate

## The checklist

1. State the marker of interest, study objectives, and working hypotheses.
  In order to understand the rationale (why this particular marker) and potential clinical applications (what is needed for this particular condition), a description of the marker of interest, study objectives, and a working hypotheses are necessary. Describe what is known on the biology of the marker, methods to detect and quantify the marker, and why the marker might be of clinical interest. A working hypothesis should be formulated as a rule in terms that can be tested statistically.
2. Describe patient characteristics and inclusion and exclusion criteria.
  Describe the clinical context of the study. Describe why a particular cohort of patients was selected and the criteria used to define the cohort, which includes inclusion and exclusion criteria. Describe clinical details of the cohort in relation to potential use of the marker of interest. As an example, when the working hypothesis is that a marker might have a different prognostic value in different stages of disease, disease stage is an essential element in the description of patient data.
3. Describe treatment details.
  Treatment (neoadjuvant, adjuvant, first line, second line, etc.) is intended to alter the disease course of a patient. Different treatment modalities might not be distributed equally between groups with or without the biomarker, and this will become an important confounding factor when correlation between outcome and marker expression is looked for. Moreover, treatment might also have an influence on marker expression if the patient was treated prior to the moment the sample was taken, which will be a confounding factor in the analysis of the impact of the biomarker. When treatment information is missing, this should be specifically stated, and in studies on marker expression in relation to treatment response, such patients should be excluded.
4. Describe the type of material used.
  Tissue samples used in retrospective studies are often convenience collections, which potentially run a serious risk of collection bias [4]. Authors should report why and how the specimens were collected and how the specimen was handled (primary tumor site or metastatic lesion, biopsy or resection, formalin-fixed paraffin-embedded or frozen tumor tissue). Where possible, data on preanalytical handling of specimens should also be given, in order to clarify potential confounding effects associated with sample condition [5]. When control samples are used, their origin should be stated as well as how they were selected. Control samples should fit into the experimental design based upon the working hypothesis, to avoid problems of unexpected differences between control and patient samples. Authors should report methodological variables as much as possible according to MISFISHIE guidelines [1]).
5. Specify how expression of the biomarker was assessed.
  A detailed description of the criteria for assessment of the presence or absence of the biomarker at tissue level allows evaluation of potential shortcomings but also will enable future researchers to reproduce the study. Some retrospective studies on classical pathological markers tend to extract data from pathology reports, instead of rereading the slides or repeating marker expression analysis for the purpose of the investigation. This runs a risk of heterogeneity between method runs or methods applied and problems of lack of inter-individual reproducibility in reading the results. This can lead to over- or underestimation of the number of patients expressing a certain marker and might introduce selection bias [6]. For purely morphological (gross or microscopical) markers, details of specimen examination, number of slides investigated, and criteria when a marker was called positive or negative should be provided.
6. Describe the number of independent (blinded) scorers and how they scored
  Visual assessment of a biomarker is an important source of variance [5]. Interpretation varies between pathologists, and biomarker data will be more robust if expression of a biomarker is scored by multiple independent observers unaware of (blinded to) the clinical parameter of interest (such as outcome). Justification of the chosen method of and criteria for (semi-)quantitative assessment should be provided in detail.
7. State the method of case selection, study design, origin of the cases, and time frame.
  Important determinants of the reliability of study results are study design and method of patient selection. Selection of cases according to clinical or pathological parameters (for example patients selected according to age, only T4 or N0 tumors) may introduce bias; therefore, details of case selection should be reported. Stating where the patients came from might provide relevant information regarding the patient population (for example a patient population from a tertiary referral hospital might differ significantly from that of a primary care center). The time frame (when cases were recruited or diagnosis was made) should also be mentioned because therapies change over time which might affect outcome.
8. State the end of the follow-up period and median follow-up time.
  In many studies, outcome is the time to an event (e.g., recurrence, death), and follow-up should be long enough to make sure that events can happen. If, for example, a biomarker is associated with the risk of dissemination, follow-up should be long enough to allow this effect to be observed. Follow-up usually ends at a specific point in time (notably this date and the median follow-up time should be stated).
9. Define all clinical endpoints examined.
  In histopathology studies, common endpoints include death and discovery of recurrence. Endpoints used in survival analysis are not always clearly defined. Analysis of time to death might include deaths from any cause or cancer-specific deaths. A clear distinction should be made between overall survival, disease-specific survival, and recurrence-free survival. Definition of parameters defining recurrence of disease should be clear. Recurrence might include local recurrence or distant metastasis or both. Local recurrence and distant metastases are two biologically different events, and the effect of a biomarker on each of these might be different. Lack of clearly defined endpoints may lead to misinterpretation of its association with a biomarker and preclude inclusion of a publication in a meta-analysis.
10. Specify all applied statistical methods.
  If the statistical methods used in a biomarker study are not clearly specified, it will be difficult or impossible for the reader to interpret the results or reproduce and validate the findings. Rather often the amount of detail provided in publications is marginal. Mathoulin-Pelissier et al. concluded that 68 % of the articles published in major journals reported insufficient information regarding the survival analysis [7].
11. Describe how interactions with other clinical/pathological variables were analyzed.
  Any seemingly interesting biomarker might interact with established clinical or pathological factors. Methods used to assess potential interactions with other variables should be described. The interactions are essential to evaluate whether or not found associations have independent value. All included variables should be clearly defined, and the choice of variables included in the study has to be justified (why variables included in the study were retained while others were left out).
12. Describe the number of patients included in the analysis and reasons for dropout.
  In retrospective biomarker studies, the number of cases included in analysis is often lower than the initial number of cases included in the study. This is mainly due to missing values, such as impossibility to (re-)evaluate staining results or missing outcome data. A solution often chosen is to restrict the analyses to samples with complete data. However, this may introduce selection bias when samples with missing data are not typical for the whole study population. It is therefore necessary to state the number of patients and events included in each analysis. Only with this information is it possible to assess the reliability of reported findings.
13. Report patient/disease characteristics (including the biomarker of interest) with number of missing values.
  A detailed description of patient characteristics and relevant histopathological parameters is needed to assess whether or not the patient cohort included in the study is representative for the condition under scrutiny. Obvious patient characteristics are age and gender, but parameters such as ethnicity, performance status, or medical history might be relevant. In case of cancer, characteristics of the lesion should include parameters defining TNM stage.
14. Describe the interaction of the factor of interest with established prognostic variables.
  As stated in point 11, a new biomarker is only useful if its effect is maintained when interaction with other prognostic factors is ruled out, or if its assessment is (quantitatively or qualitatively) superior in comparison with established prognostic variables. For evaluation of clinical value, the potential interactions between a new biomarker and established prognostic variables should therefore be reported.
15. Include at least 90 % of initial cases in univariate and multivariate analyses.
  As mentioned above, due to missing values, the number of cases included in statistical analysis is often lower than the initial number of cases included in the study. The risk of attrition bias will increase along with the proportion of cases not included in statistical analysis [6]. To minimize attrition bias, Smith et al. proposed that at least 90 % of the selected cohort should be included in the statistical analysis [8]. Sub-analyses should be avoided because of the high risk of false-positive findings due to increasingly small patient numbers.
16. Report the estimated effect (relative risk/odds ratio, confidence interval, and *p* value provided) of the biomarker in univariate analysis.
  Establishing a biomarker’s potential association with clinical outcome is the key subject in biomarker research. In univariate analysis, the relationship between the biomarker and outcome can be assessed without adjustment for additional variables. Relative risks or odds ratios with their associated confidence intervals and *p* values should be given, regardless of statistical significance. Kaplan-Meier curves should be included when illustrative, but *p* values from log rank tests should be given regardless of statistical significance. Univariate analysis should also be performed for all other variables and presented in a summarizing table.
17. Report the estimated effect (hazard ratio, confidence interval, and *p* value provided) of the biomarker in multivariate analysis.
  In multivariate analysis, the association between a biomarker and clinical outcome can be established, correcting for established prognostic variables. Authors should report which prognostic variables were included in multivariate analysis. As a rule, significant factors identified in univariate analysis should all be included. Hazard ratios with associated confidence intervals and *p* values should be given, regardless of statistical significance.
18. Report estimated effects (hazard ratio, confidence interval, and *p* values provided) of other prognostic factors included in multivariate analysis.
  Within a study, significant findings are more likely to be reported than non-significant findings. In order to prevent selective reporting bias, authors should report the effects of all prognostic factors included in the multivariable analysis; not only the marker of interest or the significant findings.
19. Interpret the results in the context of the working hypothesis elaborated in the introduction and other relevant studies; include a discussion of limitations of the study.
  Authors should critically evaluate their findings, mentioning limitations of the study and possible biases. A good discussion will allow the reader to retain a balanced perception of the importance of the results of the study.
20. Discuss potential clinical applications and implications for future research.
  The intention of biomarker studies is to develop new disease-associated parameters of which the contribution to clinical decision-making reaches beyond that of existing parameters included in the standard of care. A statistically significant association between a marker and disease outcome might seem promising, but authors should mention in the discussion which steps will be taken in order to eventually reach implementation of the marker in patient care.

## Conclusion

Adherence to guidelines on reporting, whenever possible, should facilitate a clear perception by the reader of the inherent qualities of the reported study, and we presume that it might also have a positive effect on study quality, for as much as the checkpoints we propose are already used when the study is planned. The 20 checkpoints we propose speak for themselves. We paid no attention to sample size calculations, because most histopathological studies are retrospective and based upon convenience case collections that were not set up to answer specific questions well defined before the collection was started. Checking model assumptions, standardized model making and model validation is unusual in histopathology research but might become more mainstream when this is more often performed in the context of clinical trials. For a biomarker identified in a retrospective study, we consider external validation by independent groups on separate patient cohorts of much greater value than internal validation. Our checkpoints might be of help for investigators who study tissue-based biomarkers, reviewers of manuscripts, and researchers performing meta-analyses. They should ultimately support quality improvement of histopathological studies and implementation of new findings into daily practice. We welcome feedback from the scientific community to improve on and facilitate implementation of our list of checkpoints.
